# Variations in Methods for Quantification of Crude Ash in Animal Feeds

**DOI:** 10.1093/jaoacint/qsac100

**Published:** 2022-08-19

**Authors:** Daiana F Quirino, Malber N N Palma, Marcia O Franco, Edenio Detmann

**Affiliations:** Universidade Federal de Viçosa, Department of Animal Science, Viçosa, Minas Gerais 36570-900, Brazil; Instituto Federal de Educação, Ciência e Tecnologia de Roraima, Campus Amajari, Amajari, Roraima 69343-000, Brazil; Natural Resources Institute Finland (Luke), Jokioinen FI-31600, Finland; Universidade Federal de Viçosa, Department of Animal Science, Viçosa, Minas Gerais 36570-900, Brazil; Swedish University of Agricultural Sciences, Department of Animal Nutrition and Management, Uppsala 750 07, Sweden

## Abstract

**Background:**

Crude ash is categorized as an empirical method playing an important role in the nutritional interpretation of animal feeds, allowing indirect estimation of total organic matter (OM).

**Objective:**

Our objective was to evaluate variations in laboratory procedures for crude ash quantification regarding physical parameters (i.e., time, temperature) and ashing aids and their influences on crude ash, repeatability, and discrimination power among feeds.

**Methods:**

The “control” method was based on a simple ignition time of 3 h at 550°C. The variations are briefly described: increasing ashing time to 6 h; increasing temperature to 600°C; and using two 3 h ignition cycles at 550°C with ashing aids inclusion between them: fresh air supply, fresh air supply plus distilled water, and fresh air supply plus hydrogen peroxide. A color evaluation was also performed using a colorimetric technique. Twenty-four study materials from eight different feed types were evaluated.

**Results:**

The crude ash results differed among the method variations, but a consistent decrease in the estimates was observed when liquid aids were applied, which also improved repeatability. Ash residues did not present a consistent color pattern among methods, but the residues were darker when the control method was applied.

**Conclusion:**

The method of obtaining ash residues in animal feeds based on 550°C × 3 h does not have enough robustness and may overestimate crude ash in some feeds. Adjustments in either ignition time or temperature might improve crude ash test results, but the best test results are obtained using liquid ashing aids between two ignition cycles.

**Highlights:**

The recommended method is based on the use of 550°C and two 3 h ignition cycles with water added to the ash residue between cycles.

The terms crude ash or mineral matter refer to the inorganic residue after complete oxidation of organic matter (OM; 1, 2). However, some authors have stated that this residue should be more properly termed as a residue on ignition (3) when oxidation is mostly provided by burning. Generally, the ashing procedures comprise vaporization of water and volatile compounds, and conversion of minerals into silicates, phosphates, oxides, sulfates, and chlorides, in addition to oxidizing organic substances (2).

Crude ash plays an important role in the nutritional interpretation of animal feeds by allowing indirect estimation of total OM, which encompasses all potential energy-producing compounds (4). Moreover, ash contents are also mandatory to estimate feed components quantified by difference, such as non-fiber carbohydrates and nitrogen-free extract (5, 6). Crude ash estimates have been incorporated as an important input into summative systems for the estimation of energy contents in animal diets (7–9). Actually, any bias in the crude ash estimates might decrease the accuracy of the nutritional evaluation of animal feeds, which in turn may compromise production and culminate in economic losses (3) due to inadequate diet formulation.

The standard method used to estimate % crude ash in animal feeds was first described in the early 20th century (AOAC Method **942.05**; 10, 11) and is still used worldwide as is or with some minor modifications. Briefly, a 2 g test portion is ignited at 600°C for 2 h and crude ash is reported as the residue on ignition (3).

Nevertheless, the crude ash in animal feeds could be categorized as an empirical or type I method. Thus, it would be an analytical entity that determines a value that can only be achieved in terms of the method per se (12). In this sense, there are no primary reference standards that simulate the complex associations between organic and inorganic compounds, such as those observed in feed materials. Therefore, the method itself cannot be validated for accuracy in determining the “true” value for the constituent. To minimize systematic errors (i.e., bias) among laboratories, empirical methods must be followed exactly as described in the standard manuals. Even minor variations in methodology might result in the measurement of a different constituent (13) and compromise feed interpretations and comparisons among feeds, laboratories, and analysts.

The ashing procedure can be described as the submission of a test portion to a physical binomial based on temperature and time. Despite being originally based on using 600°C, lower ignition temperatures have been suggested for the official AOAC Method since the 1940s (10, 11, 14). Excessive temperatures have been associated with systematic bias caused by the volatilization of several minerals (3, 5, 15, 16). Thiex et al. (3) revisited official AOAC Method **942.05** and suggested temperature adequacy down to 550°C. Some standards for feed analysis have followed a similar pattern of temperature adequacy, such as in ISO method 5984:2002 (17), European Commission (18), or the latest edition of the Brazilian standards for animal feed analyses (method M-001/2; 6).

On the other hand, the second variable of the physical binomial applied to crude ash quantification seems to have a more controversial pattern in the literature. Recommendations for ashing time can range from 1 h (1) to overnight (16, 19) or until a constant weight of residue on ignition is achieved (20). It is known that oxidation power into the muffle furnace results from a balance between temperature and time (21). Thus, in a logical reasoning, the higher the temperature, the shorter the ignition time, and vice versa. However, most methods seem to use modal times ranging from 2 to 3 h as they are based on temperatures between 550–600°C. In general, the use of longer ignition times seems to be an attempt to either avoid or minimize the contamination of residue by a part of the OM that is possibly more refractory to oxidation. The influence of that refractory part has been associated with dark/brown colorations of the residue on ignition (3) even after many hours of ashing.

In this sense, the introduction of ashing aids as a third element contributing to OM oxidation (i.e., besides temperature and time) has been suggested. Accordingly, Thiex et al. (3) recommended a modification to official AOAC Method **942.05**, which should encompass two 3 h ignition cycles at 550°C. The cycles are intercalated by opening the furnace door to guarantee a fresh air supply. In this case, the fresh air would serve as an oxygen supplier and would improve the oxidation of refractory organics. On the other hand, some authors have claimed that the dry ashing process may produce a heavy layer on top of the ash residue, which could hinder its complete combustion and overestimate ash content. In this case, a small amount of water could be poured over the ash residue between two ignition cycles to break up that layer (2, 3). Moreover, some variations in dry ashing methods have been based on the use of chemical substances as ashing aids (3, 18, 22, 23), probably relying on the release of free radicals to speed up oxidation.

However, despite of all the current theoretical knowledge on the dry ashing process, the recommendations regarding laboratory procedures are still highly variable (1–3, 6, 16, 18). Possibly, a “perfect” standard procedure cannot be achieved for all feed materials (3). Notwithstanding, the efficiency of variations in the procedures must be verified to assure adequate levels of method robustness while keeping an optimal ability to discriminate feed materials regarding their different chemical characteristics.

Thus, our objective was to evaluate some variations in laboratory procedures for crude ash quantification regarding physical parameters (i.e., time, temperature) and ashing aids and their influences on crude ash estimates, repeatability, and discrimination power among feed types.

## Experimental

### Location and Study Materials

All analyses were performed at the Animal Nutrition Laboratory of the Animal Science Department of the Universidade Federal de Viçosa, Viçosa, Minas Gerais, Brazil.

Eight different feed types were chosen aiming to compose a representative set regarding diets offered to beef and dairy cattle: corn silage, fresh sugarcane, sugarcane silage, Tifton-85 hay, soybean meal, corn grain, wheat bran, and dried distillers’ grains (DDG). For each feed type, three different unique materials (i.e., field replicates or primary samples) were obtained from feed industries and farms located in Minas Gerais state, Brazil. Therefore, our analytical set encompassed 24 study materials.

The high-moisture feeds (silages and fresh sugarcane) were oven-dried (55°C). Then, all dry study materials were ground in a knife mill (TE-680, Tecnal, Piracicaba, São Paulo, Brazil) to pass through a 1-mm screen sieve. The 24 study materials were then analyzed in triplicate for dry matter (DM) content (dried overnight at 105°C, method G-003/1; 6).

### Crude Ash Methods

The “control” procedure herein was based on the official method of the Brazilian National Institute of Science and Technology in Animal Science (INCT-CA; method M-001/2; 6). Briefly:

- Weigh 2.0 g as-is of the test portion into the crucible, recording the weight of crucible and test portion to the nearest 0.1 mg;
- Place the crucibles in the muffle furnace;
- Ignite in a furnace at 550°C for 3 h. The furnace must be adjusted to reach ignition temperature within 60 min. The ignition time starts counting after ignition temperature is achieved;
- Allow the furnace to cool below 200°C, but stay above 150°C. Then transfer crucibles to a desiccator; and
- Cool to room temperature and weigh, recording the weight to the nearest 0.1 mg.

For all procedures, the crucibles (5 cm diameter and 30 mL volume) were previously washed in running water, ashed for 3 h at 550°C, and handled and weighed as described above. The same digital muffle furnace was used for all procedures (Fornos Magnus, Belo Horizonte, Minas Gerais, Brazil).

The following variations on the basic method were evaluated:

1. Increased ignition temperature: all procedures were performed as previously described for the control, but the temperature was increased to 600°C (2, 10);
2. Increased ashing time: all procedures were performed as previously described for the control, but ashing time was increased to 6 h. This specific time was chosen to allow a direct comparison with method variations that included ashing aids;
3. Using fresh air as an ashing aid: the procedures were adapted from Thiex et al. (3). The following modifications were added to the control procedure:
(d)
Allow the furnace to cool below 200°C and open the door to ensure a fresh air supply (1-2 min);
(e)
Reignite in a furnace at 550°C for 3 h. The ignition time starts counting after ignition temperature is achieved;
(f)
Allow the furnace to cool below 200°C, but stay above 150°C. Then transfer crucibles to a desiccator; and
(g)
Cool to room temperature and weigh, recording the weight to the nearest 0.1 mg;
4. Using fresh air and water as ashing aids: the procedures were adapted from Thiex et al. (3) and, besides fresh air supply, they aim at breaking the superficial layer of residue on ignition and allowing more efficient ashing of the bottom layer in the second ignition cycle. The modifications of procedures compared to the control were:
(d)
Allow the furnace to cool below 100°C and open the door;
(e)
Carefully add a few mL of distilled water to the residue to break it up;
(f)
Reignite in a furnace at 550°C for 3 h. The ignition time starts counting after ignition temperature is achieved;
(g)
Allow the furnace to cool below 200°C, but stay above 150°C. Then transfer crucibles to a desiccator; and
(h)
Cool to room temperature and weigh, recording the weight to the nearest 0.1 mg;
5. Using fresh air and hydrogen peroxide as ashing aids: the procedures were the same as described in the latter variation, except that water was replaced by hydrogen peroxide PA (35% or 130 volume). In this case, besides breaking up the upper layer, we hypothesized the hydrogen peroxide decomposition releases free radicals, which could speed up the oxidation of the residual OM.

Each ashing run contained all 24 study materials. We performed three ashing runs (*n* = 3) for each method, totaling the evaluation of 432 aliquots (i.e., test portions).

Following the recommendations of Thiex et al. (3), we also performed a color evaluation of residues on ignition. Due to the small masses, the residues from the three replicates were pooled and scored for color pattern (L* for lightness, a* for redness, and b* for yellowness), using a Hunter MiniScan EZ colorimeter (4500 L; Hunter Associates Laboratory Inc., Reston, VA, USA). Those coordinates were then converted into RGB (i.e., red, green, and blue) coordinates using Coloroid Professional Color Plan Designer software. After that, RGB coordinates were organized in an Excel plan in which each cell was filled with the corresponding solid color. For some materials, the mass of residues on ignition was not sufficient to allow the color evaluation.

### Calculations and Statistical Analysis

The calculation of crude ash was performed according to the following equation:

(1) $${\%\text{CA}}_{\text{DM}}=\frac{R-T}{W\times \text{DM}}\times 100,$$

where %CA_DM_ = crude ash as a percentage of DM; R = weight of crucible + residue on ignition (g); T = tare (empty) weight of crucible (g); W = weight of test portion (g, as-is); and DM = dry matter content of the sample (g/g).

The crude ash results were analyzed according to the model:

(2) $$Y_{\text{ijkl}}=\mu +F_{i\;}+S_{\left(i\right)j}+M_{k}+{\text{FM}}_{\text{ik}}+\varepsilon_{\text{ijkl}},$$

where Y_ijkl_ = crude ash obtained in the test portion l taken from study material j of feed type i and evaluated through method k; µ = general constant; F_i_ = fixed effect of feed type i; S_(i)j_ = random effect of study material j nested to feed i assumed to be NIID (0, $\sigma_{S/F}^{2}$); M_k_ = fixed effect of method k; FM_ik_ = fixed effect of interaction between feed type i and method k; and ε_ijkl_ = random error assumed to be NIID (0, $\sigma_{\varepsilon }^{2}$).

When necessary, means were grouped using Fisher’s multiple comparison procedure.

After the first analysis of variance, data was analyzed again in an independent way for each method according to the model:

(3) $$Y_{\text{ijk}}=\mu +F_{i\;}+S_{\left(i\right)j\;}+\varepsilon_{\text{ijk}},$$

where Y_ijk_ = crude ash obtained in the test portion k taken from the study material j of feed type i; µ = general constant; F_i_ = random effect of feed type i assumed to be NIID (0, $\sigma_{F}^{2}$); S_(i)j_ = random effect of study material j nested to feed type i assumed to be NIID (0, $\sigma_{S/F}^{2}$); and ε_ijk_ = random error assumed to be NIID (0, $\sigma_{\varepsilon }^{2}$).

From the adjustment of the model described in equation (3), the following relative standard deviations were estimated for each method variation:

(4) $$r=\frac{\sqrt{{\hat{\sigma }}_{\varepsilon }^{2}}}{\bar{Y}}\times 100,$$

(5) $${\text{RSD}}_{F}=\frac{\sqrt{{\hat{\sigma }}_{F\;}^{2}}}{\bar{Y}}\times 100,$$

(6) $${\text{RSD}}_{S}=\frac{\sqrt{{\hat{\sigma }}_{S/F}^{2}}}{\bar{Y}}\times 100,$$

where r = repeatability (%); ${\hat{\sigma }}_{\varepsilon }^{2}$ = estimate of error variance [(% DM)^2^]; $\bar{Y}$ = average crude ash (% DM); RSD_F_ = relative standard deviation among feed types (%); ${\hat{\sigma }}_{F}^{2}$ = estimate of the variance among feed types [(% DM)^2^]; RSD_S_ = relative standard deviation among study materials (%); and ${\hat{\sigma }}_{S/F}^{2}$ = estimate of the variance among study materials within feed types [(% DM)^2^].

Although some reference values have been established regarding reproducibility (24, 25), it has been difficult to define adequate limits for repeatability. We know that the reproducibility evaluation cannot be performed in our work, as all procedures were performed in one single laboratory. However, we adopted a more functional approach to assess repeatability from the expected reproducibility value, which was calculated as:

(7) $$Re=2\times C^{-0.15},$$

where Re = expected reproducibility (%) and C = average content of crude ash (g/g DM).

According to Horwitz (24), repeatability should ordinarily be one-half to two-thirds of reproducibility. From this, we assumed that the expected conventional limits for repeatability could be established as:

(8a) $$L_{c}=0.50\times Re,$$

(8b) $$U_{c}=0.67\times Re,$$

where L_c_ and U_c_ are the lower and upper limits for the expected conventional repeatability (%).

Equation (8) is based on the assumption that reproducibility behaves exactly as expected [Equation (7)]. However, in general, a method is considered reproducible if the actual reproducibility falls between one-half and twice the expected reproducibility (24, 25). From this, the conventional limits for repeatability may be adjusted to a tolerable range according to:

(9a) $$L_{t}=0.50\times 0.50\times Re=0.25\times Re,$$

(9b) $$U_{t}=0.67\times 2.00\times Re=1.34\times Re,$$

where L_t_ and U_t_ are the lower and upper limits for the expected tolerable repeatability (%).

From equations (8) and (9) we were able to understand how adequately the method variations behaved regarding precision/repeatability.

All statistical evaluations were performed using the GLIMMIX procedure of SAS 9.4. The variance components were estimated according to the restricted maximum likelihood method. Statistical significances were declared at *P* < 0.05.

## Results

There was an interaction (*P* < 0.01) between feed types and methods on the crude ash. The slicing of this effect indicated that for most feed types (corn grain, DDG, wheat bran, grass hay, and fresh sugarcane) the methods did not affect (*P* ≥ 0.23) the values of residues on ignition (Table 1). However, differences among methods occurred (*P* < 0.01) for soybean meal, corn silage, and sugarcane silage.

**Table 1.** Least-square means for the residue on ignition (% of DM) in different feed types according to the crude ash method

		Method^a^							
	Temperature, °C	550	600	550	550	550	550		
			
	Time, h	3	3	6	3 + 3	3 + 3	3 + 3		
			
Feed type	Ashing aid	—	—	—	Fresh Air	Air + H_2_O	Air + H_2_O_2_	SEM	*P*-value
Corn grain		1.18	1.18	1.17	1.13	1.15	1.13	0.645	0.689
DDG		3.06	3.00	3.11	3.03	3.04	3.04		0.230
Soybean meal		6.67a	6.49b	6.49b	6.40c	6.33c	6.32c		<0.001
Wheat bran		6.48	6.49	6.44	6.43	6.47	6.45		0.679
Grass hay		5.39	5.42	5.41	5.41	5.33	5.35		0.244
Corn silage		4.71a	4.72a	4.70a	4.67a	4.59b	4.57b		<0.001
Fresh sugarcane		2.25	2.15	2.22	2.22	2.17	2.20		0.252
Sugarcane silage		4.20ab	4.24a	4.10c	4.18ab	4.12bc	4.11bc		0.003
Overall		4.24a	4.21b	4.20b	4.18b	4.15c	4.15c	0.228	<0.001

a Means in a row followed by different letters differ at *P* < 0.05.

The comparisons among methods within each feed type were generally different from each other and some overlaps were observed (Table 1). For soybean meal, the general pattern indicated that just increasing either time or temperature decreased (*P* < 0.05) crude ash in comparison to the control method. However, a more consistent decrease compared to the control (*P* < 0.05) was seen when ashing aids were applied. All ashing-aid variations clustered together (*P* > 0.05). On the other hand, the mean comparisons for corn silage indicated that increasing neither temperature nor ashing time was sufficient (*P* > 0.05) to decrease crude ash in comparison to the control. Effective decreases were only obtained (*P* < 0.05) when liquid ashing aids were used, which did not differ from each other (*P* > 0.05). The mean comparisons for sugarcane silage were very uninformative and a clear pattern could not be extracted from them.

**Figure 1.**
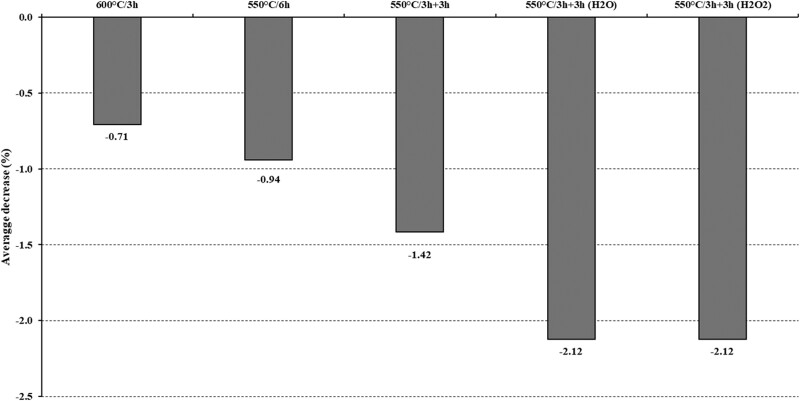
Average percentage decrease in residue on ignition according to the crude ash methods in relation to the control method (i.e., 550°C × 3 h).

On average, the overall mean comparisons indicated that increasing either temperature or ashing time caused a consistent decrease in crude ash when compared to the control method (*P* > 0.05, Table 1, Figure 1). In terms of ashing aids, the simple introduction of fresh air was not enough to decrease (*P* > 0.05) crude ash in comparison to simply increasing the temperature or time. However, the use of liquid ashing aids caused an additional decrease (*P* < 0.05) in crude ash compared to the other methods.

The RSDs among feed types and study materials behaved similarly among methods (Table 2) and repeatability was found within the tolerable boundaries for all methods. However, when liquid ashing aids were added, the repeatability was slightly improved.

**Table 2.** Descriptive statistics of random variabilities for the residue on ignition according to the crude ash method

		Method					
	Temperature, °C	550	600	550	550	550	550
	
	Time, h	3	3	6	3 + 3	3 + 3	3 + 3
	
Item	Ashing aid	—	—	—	Fresh Air	Air + H_2_O	Air + H_2_O_2_
RSD, %							
Among feed types		43.9	44.0	43.4	43.6	43.6	43.6
Among study materials		26.5	26.4	27.1	26.6	26.6	26.5
Repeatability (r)		2.13	2.73	2.80	2.12	1.50	1.24
Expected reproducibility, %		3.21	3.22	3.22	3.22	3.22	3.22
Expected limits for r, %^a^							
Conventional		1.61–2.15	1.61–2.16	1.61–2.16	1.61–2.16	1.61–2.16	1.61–2.16
Maximum tolerable		0.80–4.31	0.80–4.31	0.80–4.31	0.80–4.31	0.81–4.32	0.81–4.32

a *See* equations (7), (8), and (9) for more details.

The color evaluation was performed fully only for four feed types (Table 3). For the others, we faced constraints to obtain residues masses in sufficient quantities to allow an adequate color measurement by the colorimetric method. However, for the feed materials we had, there was no consistent pattern among methods. However, one specific point in the pattern seemed consistent enough to draw some inference. The residues on ignition were darker when the control method was applied, which is in line with the crude ash results. No systematic differences in the color pattern were verified among the other methods.

**Table 3.** Color pattern of the residue on ignition in different feed types according to the crude ash method

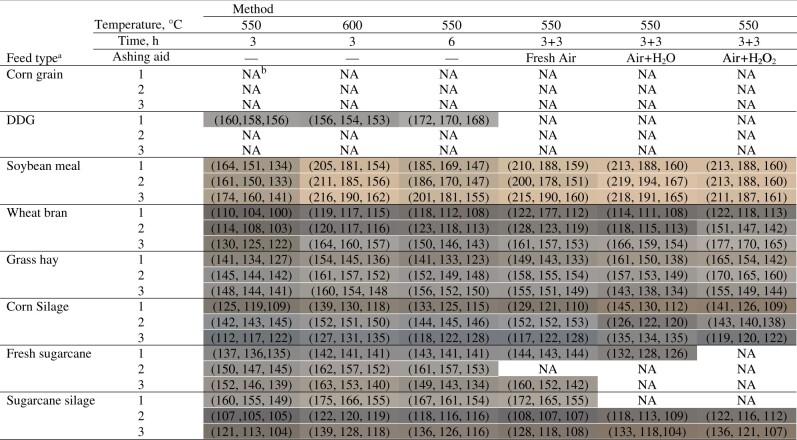

a The numbers 1 to 3 indicate the different study materials (i.e., field samples) evaluated within each feed type.

b NA = Not available. The residue on ignition was insufficient for the color evaluation using the colorimeter.

## Discussion

The crude ash in feeds is assessed as the residue on ignition after a complete OM decomposition in a muffle furnace using a time × temperature binomial. Due to their empirical nature, ashing methods have been revised (3, 26) aiming at minimizing their associated biases and seeking a balance between complete oxidation and loss of volatile minerals. In general, biases might be generated from incomplete OM decomposition, volatilization of some mineral compounds under specified temperatures, or inappropriate method application (1, 3, 16, 26).

Despite being independent physical parameters, time and temperature do not act independently, and interactions between them will define the amount of crude ash obtained from a specific feed material (21). Once the adequate temperature is established, adjustments on ignition time would provide some fine-tuning on the analytical entity’s estimates and vice versa. On the other hand, temperatures below the minimum required for adequate ignition of the OM may not be compensated by extending ignition time. An equilibrium between the physical parameters of the process must be achieved to allow an appropriate quantification of crude ash in feeds.

The results here confirmed that increases in either ignition time or temperature led, on average, to decreases in the % crude ash. They bring evidence that the physical binomial 550°C × 3 h is not the best option to quantify the crude ash in feed materials. This was the only clear pattern to emerge from the colorimetric evaluation of residues on ignition. Some feed types were not sensitive to the variations in the physical parameters; however, others were. Thus, to achieve more adequate robustness, the chosen method should focus on the feed types that are particularly sensitive to the method.

An ideal ignition temperature should be as low as possible to reduce volatile compound losses, yet high enough to ensure total carbon loss (23). A recurrent issue associated with excessive temperatures is the loss of minerals through volatilization (26, 27). From this, it could be speculated that increasing the temperature to 600°C could have decreased ash content due to increased volatilization. However, the decreased ash content obtained by extending ashing time seems to allow a different explanation for that. Considering that the simple extension of either time or temperature led to the same average decrease in % crude ash, the most probable cause was an improvement in the elimination of some refractory OM, rather than an increase in volatilization. Once more, the inadequacy of the control procedure is evident.

However, despite the aforementioned likely improvement the % crude ash was, on average, further reduced with the use of liquid ashing aids. In this particular case, any loss increased by volatilization has no physical or chemical reasons to occur. However, the pattern obtained with the different ashing aids was not consistent among feed types.

Despite clustering along with the liquid ashing aids for soybean meal and sugarcane silage, on average, the simple fresh air supply was not enough to reduce the crude ash to the same levels observed when liquid aids were applied. It has been stated that a fresh air supply between two ignition cycles could renew the oxygen supply inside a muffle furnace. Consequently, it could improve the release of carbon that might remain in the sample after the first ignition cycle (3, 28). However, such a pattern did not correspond to what was observed here.

Further improvements in crude ash estimates were obtained only when liquid ashing aids were added between the two ignition cycles. During the ashing procedure, a heavy layer might be formed on the top of the residue interfering with carbon release (2). Then, adding liquid aids between ignition cycles might improve OM decomposition by mechanical action, crushing the crust eventually formed in the previous ignition cycle and improving the degradation of refractory compounds in the second ignition cycle (27). Hydrogen peroxide might accelerate OM combustion in dry ashing methods (23). The decomposition of hydrogen peroxide basically occurs when its highly unstable oxygen–oxygen bond is broken, which can release free radicals, such as hydroxyl and hydroperoxyl (29). Those are highly reactive and may improve OM decomposition. However, we did not observe differences between using water and hydrogen peroxide as ashing aids, indicating that their effects were similar and most likely associated with the physical breakdown of the heavy layer on the top of the ash residue, allowing better oxidation of material below the crust during the second ignition cycle. Considering the similarity of both liquid ashing aids, water is recommended, mainly due to its lower cost and ease of use.

Several authors have considered that complete ashing is reached when heating is continued until the residue on ignition achieves a uniform color and is free from unburned particles (30, 31). In this sense, the color should be as light as possible (i.e., white, light gray; occasionally reddish or green). Accordingly, if the residue is dark or brown, it could indicate an undesirable carbon presence (3, 28, 31, 32).

However, any visual color evaluation can be biased, as it depends on the subject's judgment. There is no standard color chart available for color evaluations of ash residue, which makes visual evaluations subjective and imprecise. Color scoring would be the result of a light interaction between the object and the observer’s eyes, and can also vary according to the ambient lighting (33). That is the reason why we decided to perform a color evaluation using a colorimetric technique, which would avoid any subjectivity when scoring the residue coloration. In general, the control method (i.e., 550°C × 3 h) presented the darkest residues, but no clear pattern among feeds was observed for the other methods.

Despite confirming the inadequacy of the control method, the inconsistent pattern among methods indicates that ash color seems to be more a characteristic of the feed material itself rather than particularly useful information to evaluate the ashing quality. Even though improvements in crude ash had been obtained using liquid ashing aids, none of the ash residues showed a color pattern close to white or light gray. Thus, the results here obtained agreed with St. John (30), who stated that neither macroscopic observations nor an analyst’s judgment for carbon presence in crude ash are useful in determining the optimal ashing methodology.

Precision in our study was represented by repeatability, which is also known as within-laboratory variation (24). Repeatability is based on random residual variance, and lower values indicate a more reliable procedure for feed evaluation (34). Repeatability limits were proposed and calculated based on the expected reproducibility and all method variations exhibited repeatability within tolerable limits. However, the use of liquid ashing aids improved repeatability. Such a pattern reinforces our previous discussion. Besides overestimating crude ash, the OM retained in the bottom layer of ash residues seems to be variable among test portions. Thus, the action of the liquid ashing aids also improves the precision of the procedures by decreasing random variation among replicates.

In terms of feed analysis, an ideal method must also be able to allow adequate discrimination among and within feed types. That characteristic is expressed here by the RSDs among feed types and study materials. Unlike repeatability, it is desirable that those RSDs be maximized under any given evaluation. Hence, the capability to discriminate feeds with different characteristics, as well as to cluster similar feeds would be surely guaranteed. Regarding those requisites, all methods evaluated herein performed similarly.

## Conclusions

The method of obtaining residues on ignition in animal feeds based on the binomial 550°C × 3 h does not have sufficient robustness and may overestimate crude ash in some feeds. Adjustments in either ignition time or temperature appear to improve crude ash test results, but the best results are obtained using liquid ashing aids between two ignition cycles. The recommended method is based on the use of 550°C and two 3 h ignition cycles with water added to the ash residue between cycles.

## References

[1] Ismail B.P. (2017) in Food Analysis Laboratory Manual, Nielsen S.S. (Ed.), Springer, New York, NY, pp 117–119. doi:10.1007/978-3-319-44127-6_11

[2] Liu K. (2019) Algal Res. 40, 101486. doi:10.1016/j.algal.2019.101486

[3] Thiex N. , Novotny L., Crawford A. (2012) J. AOAC Int. 95, 1392–1397. doi:10.5740/jaoacint.12-129 23175971

[4] Souza M.A. , Detmann E., Batista E.D., Franco M.O., Valadares Filho S.C., Pina D.S., Rocha G.C. (2017) Rev. Bras. Saúde Prod. Anim. 18, 62–75. doi:10.1590/s1519-99402017000100007

[5] Pojić M. , Mastilovic J., Palic D., Pestoric M. (2010) Food Chem. 123, 800–805. doi:10.1016/j.foodchem.2010.05.013.

[6] Detmann E. , Costa e Silva L.F., Rocha G.C., Palma M.N.N., Rodrigues J.P.P. (2021) Métodos Para Análises de Alimentos. 2nd Ed. Suprema, Visconde do Rio Branco, Brazil

[7] Tedeschi L.O. , Fox D.G., Doane P.H. (2005) Prof. Anim. Sci. 21, 403–415. doi:10.15232/S1080-7446(15)31238-9

[8] Detmann E. , Silva T.E., Valadares Filho S.C., Sampaio C.B., Palma M.N.N. (2016) in Nutrient Requirements of Zebu and Crossbred Cattle BR-CORTE, 3rd Ed. Valadares Filho S.C., Costa e Silva L.F., Gionbelli M.P., Rotta P.P., Marcondes M.I., Chizzotti M.L., Prados L.F. (Eds), Suprema, Visconde do Rio Branco, Brazil, pp 89–126

[9] NRC (2021) Nutrient Requirements of Dairy Cattle, 8th Ed., National Academies Press, Washington, DC. doi:10.17226/9825 38386771

[10] St. John J.L. (1942) J. AOAC Int. 25, 857–863. doi:10.1093/25.4.857

[11] St. John J.L. (1943) J. AOAC Int. 26, 220–226. doi:10.1093/jaoac/26.2.220

[12] Codex Alimentarius Commission (2018) Procedural Manual, 26th Ed., Food and Agriculture Organization of the United Nations, Rome, Italy

[13] Mertens D.R. (2003) J. Anim. Sci. 81, 3233–3249. doi:10.2527/2003.81123233x 14677881

[14] St. John J.L. (1940) J. AOAC Int. 23, 620–636. doi:10.1093/jaoac/23.3.620

[15] Isaac R.A. , Jones J.B. Jr (1972) Commun. Soil Sci. Plant Anal. 3, 37–41. doi:10.1080/00103627209366375

[16] Marshall M.R. (2010) in Food Analysis, Nielsen S.S. (Ed), Springer, New York, NY, pp 105–115. doi:10.1007/978-1-4419-1478-1_7

[17] ISO 5984:2002: Animal Feeding Stuffs-Determination of Crude Ash, International Organization for Standardization Oxford University Press, Stockolm, Sweden

[18] EC (2009) Commission Regulation (EC) No 152/2009 of 27 January 2009, laying down the methods of sampling and analysis for the official control of feed, https://eur-lex.europa.eu/legal-content/EN/TXT/?uri=CELEX:32009R0152 (accessed on May 15, 2022)

[19] Van Soest P.J. , Robertson J.B. (1985) Analysis of Forages and Fibrous Foods, Cornell University, Ithaca, NY

[20] AACC (1999) International Approved Methods of Analysis, 11th Ed., AACC International, St. Paul, MN, **Method 08-03.01**. doi:10.1094/AACCIntMethod-08-03.01

[21] Matthiessen M.K. , Larney F.J., Selinger L.B., Olson A.F. (2005) Soil Sci. Plant Anal. 36, 2561–2573. doi:10.1080/00103620500257242

[22] Cecchi H.M. (2003) Fundamentos Teóricos e Práticos em Análise de Alimentos, 2nd Ed., Editora da Unicamp, Campinas, Brazil. doi:10.7476/9788526814721

[23] Pojić M. , Kravić S., Stojanović Z. (2015) in Handbook of Food Analysis, 3rd Ed., Nollet M.L.L., Toldrá F. (Eds), CRC, Boca Raton, FL, pp 275–296

[24] Horwitz W. (1982) Anal. Chem. 54, 67–76. doi:10.1021/ac00238a002

[25] Horwitz W. , Albert R. (2006) J. AOAC Int. 89, 1095–1109. doi:10.1093/jaoac/89.4.1095 16915851

[26] Rowan C.A. , Zajicek O.T., Calabrese E.J. (1982) Anal. Chem. 54, 149–151. doi:10.1021/ac00238a047

[27] Mader P. , Száková J., Curdová E. (1996) Talanta 43, 521–534. doi:10.1016/0039-9140(95)01793-3 18966515

[28] Hoenig M. (2005) in Encyclopedia of Analytical Science, 2nd Ed., Worsfold P., Townshend A., Poole C. (Eds), Elsevier, Oxford, UK, pp 131–136. doi:10.1016/b0-12-369397-7/00537-9

[29] Mikutta R. , Kleber M., Kaiser K., Jahn R. (2005) Soil Sci. Soc. Am. J. 69, 120–135. doi:10.2136/sssaj2005.0120

[30] St. John J.L. (1942) J. AOAC Int. 25, 969–972. doi:10.1093/jaoac/25.4.969

[31] Park Y.W. (1996) in Handbook of Food Analysis, Nollet L.M.L. (Ed), Marcel Dekker, New York, NY, pp 59–92

[32] Park Y.W. , Bell L.N. (2004) in Handbook of Food Analysis: Physical Characterization and Nutrient Analysis, Nollet L.M.L. (Ed), Marcel Dekker, New York, NY, pp 55–82. doi:10.1201/b11081-5

[33] Choudhury A.K.R. (2015) Visual Measurement of Colour, Colour Comparison and Management, Vol. 2, 1st Ed., Woodhead Publishing, Sawston, UK. doi:10.1016/C2014-0-01832-1

[34] Silva T.E. , Detmann E., Camacho L.F., Saliba E.O.S., Palma M.N.N., Valadares Filho S.C. (2017) Arq. Bras. Med. Vet. Zootec. 69, 1635–1644. doi:10.1590/1678-4162-9096

